# Morphogens and Cell-Derived Structures (Exosomes and Cytonemes) as Components of the Communication Between Cells

**DOI:** 10.3390/ijms26030881

**Published:** 2025-01-21

**Authors:** Stavros Chideriotis, Alkmini T. Anastasiadi, Vassilis L. Tzounakas, Sotirios P. Fortis, Anastasios G. Kriebardis, Serena Valsami

**Affiliations:** 1 Hematology Department, Elena Venizelou Hospital, 11521 Athens, Greece; stauroschyder1972@gmail.com; 2 Department of Biochemistry, School of Medicine, University of Patras, 26504 Patras, Greece; aanastasiadi@upatras.gr (A.T.A.); vtzounakas@upatras.gr (V.L.T.); 3 Laboratory of Reliability and Quality Control in Laboratory Hematology (HemQcR), Department of Biomedical Sciences, School of Health & Caring Sciences, University of West Attica (UniWA), 12243 Egaleo, Greece; sfortis@uniwa.gr (S.P.F.); akrieb@uniwa.gr (A.G.K.); 4 Hematology Laboratory, Blood Bank, Aretaieion Hospital, National and Kapodistrian University of Athens, 11528 Athens, Greece

**Keywords:** morphogens, exosomes, cytonemes, cellular communication

## Abstract

Morphogens, which are non-classical transcription factors, according to several studies, display a crucial role in tissue patterning, organ architecture establishment, and human disease pathogenesis. Recent advances have expanded the morphogen participation to a wide range of human diseases. There are many genetic syndromes caused by mutations of components of morphogen signaling pathways. The aberrant morphogen pathways also promote cancer cell maintenance, renewal, proliferation, and migration. On the other hand, exosomes and their application in the biomedical field are of evolving significance. The evidence that membrane structures participate in the creation of morphogenic gradience and biodistribution of morphogen components renders them attractive as new therapeutic tools. This intercellular morphogen transport is performed by cell-derived structures, mainly exosomes and cytonemes, and extracellular substances like heparan sulphate proteoglycans and lipoproteins. The interaction between morphogens and Extracellular Vesicles has been observed at first in the most studied insect, Drosophila, and afterwards analogous findings have been proved in vertebrates. This review presents the protagonists and mechanisms of lipid-modified morphogens (Hedgehog and Wnt/β-catenin) biodistribution.

## 1. Morphogens

### 1.1. Morphogen Definition

A morphogen is a particular type of signaling molecule governing the pattern of tissue development and the location of various specialized cell types within a tissue [1]. It spreads from a localized source and establishes a concentration gradient across a developing tissue. The action of these transcription substances is to promote different cellular responses in a concentration-dependent manner [2]. Morphogen concentration gradients induce the biogenesis of different cell types and their placement in a tissue in a three-dimensional determined order [3].

### 1.2. Historical Background

The morphogen model of tissue patterning is promoted by a long history in the field of biology. In the beginning of the 20th century, Lewis Wolbert defined the morphogen characteristics and function with his known “French flag” [4] model which explained the mechanism of a tissue separation into domains of different target gene transcription (analogous to the colors of the “French flag”). The first defined morphogen is Bicoid [5], which is a transcription factor observed in different concentrations in the Drosophila syncytial embryo. Later, Stephen Cohe described how a produced signaling substance Decapentaplegic (the Drosophila homolog of TGF-B) [6] functions as a morphogen in Drosophila. Following studies, related to the development of animals, demonstrated the huge significance of morphogens in driving animal tissue patterning and growth [7].

### 1.3. Morphogen Biological Role

The mechanism by which morphogens induce tissue patterning has been studied. The population of the cells is divided into different types, according to the distance of the origin of morphogens [7]. Cells far from the restricted source of the morphogen production will be influenced by low concentrations of morphogens, and only low threshold target genes will be transcribed. In contrast, cells near the restricted origin of morphogen production will receive high levels of a morphogen and both low- and high-threshold target gene transcription will take place [7].

Another function of morphogens regards the specific spatial placement of each cell in every tissue during embryogenesis, in order to establish functional organogenesis [8]. Derangement of this function results in several organ malformations such as polydactyly, syndactyly, facial abnormalities, holoprocencephaly, spinal-rib disorders, and Pallister Hall syndrome [9]. It also participates in the development of neurodegenerative diseases, bipolar disorders, serum negative rheumatoid arthritis and carcinogenesis later in life [10].

Hedgehog and Wnt/β catenin pathways are involved in the processes of tissue patterning, whereby cells adopt certain cell fates and organize into a tissue, and polarity establishment, the asymmetric arrangement of cellular proteins that guides the organization and orientation of distinct tissue layers [11].

The Hedgehog Family of intercellular signaling proteins activate a highly conserved signaling pathway that plays an essential role in developmental and tissue patterning during embryogenesis, and it is also important in adult tissues homeostasis and regeneration of adult tissues. The three mammalian proteins (Sonic Hedgehog (Shh), Indian hedgehog (Ihh), and Desert hedgehog (Dhh)) share a common signaling pathway which is initiated by the binding of these proteins to their receptor protein Patched + (PTCH-1), thereby allowing the G protein-coupled receptor smoothened (SMO) to be phosphorylated and trigger the downstream events [12].

The canonical WNT pathway is highly conserved and activated through the binding of extracellular Wnt ligands (Wnt 3a, Wnt-1, Wnt 5a) to the Wnt receptors Frizzled (Fzd) and LRP 5/6. The cytoplasmic segment mainly includes β-catenin. The nuclear segment mainly includes β-catenin, which translocates to the nucleus. Once activated, the typical Wnt pathway induces the stability of β-catenin and transfers it to the nucleus promoting the expression of genes involved in cell proliferation, survival, differentiation and migration [13].

## 2. Sonic Hedgehog Proteins and Wnt/β-Catenin Pathway

### 2.1. Sonic Hedgehog Proteins’ Biological Role

The biological significance of the Sonic hedgehog (Shh) components has been reported from tooth and skeletal shaping to neuronal development [14]. Shh governs anatomy characteristics such as lateral asymmetry limb shaping, digit number and their location, and muscle precursors’ differentiation and maintenance [14]. Shh proteins are critical in hematopoietic cell stem cell proliferation [15], while they also promote the delivery of endothelial stem cells from bone marrow to injured tissues via blood circulation, to induce wound healing by re-establishing normal tissue architecture [16].

In addition, the significant function of Shh component signaling in the developmental process is confirmed by sterol anomalies that influence normal growth [17]. In Smith Lemli Opitz syndrome, the Shh pathway function is suppressed either by insufficient sterolation of the Shh component or by decreased responsiveness of the target cell to Shh signaling components [18]. Mutated Shh proteins lead to skeletal anomalies, holoprosencephaly, and other facial skeletal disorders [18].

**Figure 1:**
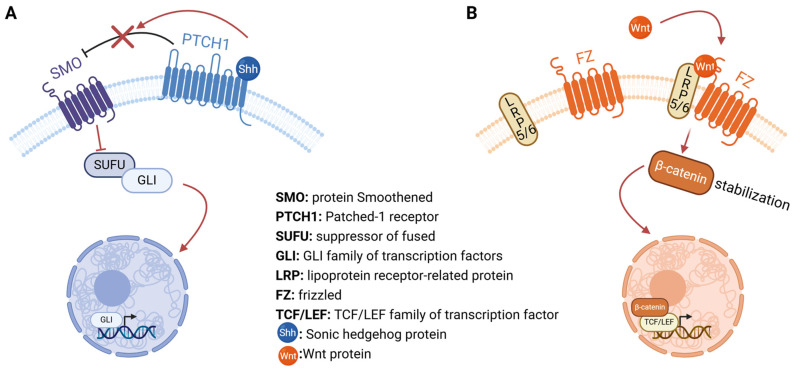
Graphical presentation of the Sonic hedgehog (Shh; (**A**)) and Wnt-β-catenin (**B**) signaling pathways. Shh inhibits the inhibitory action of PTCH1 upon the SMO protein, releasing GLI transcription factor from the SUFU protein and allowing it to enter the nucleus to upregulate the transcription of gene targets. On the other hand, Wnt protein binds to its receptor, which is comprised of LRP and FZ proteins. The activation of this complex stabilizes β-catenin by suppressing its proteasomal degradation, allowing it to translocate to the nucleus, where it inactivates the repressing TCF/LEF transcription factors. Created in BioRender.com/d65k512.

There are also some congenital disorders induced by Shh gain of function mutations. These syndromes are caused either by a mutation to Smoothened (SMO)—a G-protein-coupled receptor—which abrogates the inhibitor function of Patched 1 protein (PTCH-1) [19], or, more commonly, from a PTCH-1 mutation which negatively affects it by downregulating the action of SMO [19] (Figure 1A). Gorlin Syndrome Disorder is linked to increased activity of the Shh pathway, because of a mutation in one of the PTCH-1 genes. The most common signs of this congenital syndrome include basal cell carcinoma, brain tumors, (meningioma or medulloblastoma), and disorders of the spine, ribs, and skull [19].

### 2.2. Wnt/β-Catenin Pathway Signaling Functions

The Wnt/β-catenin canonical signaling pathway promotes self-renewal of embryonic stem cells, and induces pluripotency in them, with this function having clinical utility in the management of degenerative diseases [20] (Figure 1B). The activation of the Wnt signaling pathway induces osteogenesis of mesenchymal stem cell pools in a concentration-dependent manner, which means that low concentration of Wnt-B-catenin promotes proliferation and stemness of mesenchymal stem cells, while high levels of Wnt signaling pathway components stimulate osteogenesis [21,22]. Wnt-B-catenin modulates stem cell differentiation in the intestine. Abrogation of Wnt by D.K.K.-1 (a Wnt signaling inhibitor) leads to the absence of intestine crypt development, which represent the self-replenishment tissues of the intestine [22]. On the other hand, stimulation of the Wnt signaling pathway upregulates crypt progenitors [22]. Wnt/B-catenin activation stimulates the proliferation of hematopoietic progenitors. Blocking of Wnt3a downregulates hematopoietic stem cells, while excessive activity of the Wnt signaling pathway favors the proliferation of these stem cells.

Regarding hematologic malignancies, it has been shown in vitro that the inhibition of β-catenin through ectopic expression of axin decreases the replating capacity of leukemic cells, suggesting that human chronic myelogenous leukemia (CML) precursors are dependent on Wnt signaling for growth and renewal. Accordingly, it has been shown that the progression of CML using a mouse model in vivo is also critically dependent on intact β-catenin. On the contrary, in the absence of β-catenin, B acute lymphoblastic leukemia (ALL) formation is not impaired, and this could be attributed to the different cells of origin of ALL and CML. Indeed, it has been proposed that the cell of origin for CML is a stem cell [23]. Furthermore, the Wnt/β-catenin pathway drives cell fate, cell death, senescence, and cancer cell metastasis [24].

In addition, it is worth mentioning that in the natural setting, Wnt signaling is significant for the creation of hair cells and that the inhibition of β-catenin in the epithelium of the skin results in hair follicle stem cells’ complete loss [25].

## 3. Exosomes

### 3.1. Biolocical Aspects and Historical Background

It has been more than 50 years since the first observation and characterization of EVs as ”platelet dust”, and about 40 years since the description of their secretion from cells via endocytic pathways in sheep reticulocytes [26]. At first, the function of exosomes was unclear. Gradually, their application in the diagnostic field, drug delivery, and regenerative medicine methods, and their utility as viral and cancer vaccines led to their recognized biological role [27]. Simultaneously, researchers gained a detailed understanding of their biosynthesis, and their role in intercellular communication became clear. Exosomes’ molecular cargo is variable, and it is dependent on the cell type which secretes them and the needs of target cells [27]. Nevertheless, a plethora of exosomal proteins (transmembrane: tetraspanin, flotillin; cytosolic: heat shock proteins HSP70, HSP90, annexins, TSG01 and MHC) [27] are used as exosome markers, along with the small GTPases of the Rab family. Multiple biological functions of exosomes have been observed, including participation in angiogenesis, inflammation, malignancy progression, cell proliferation and differentiation, and involvement in morphogen delivery. In addition, their significant role as biomarkers in health and pathological states is well-established [28].

### 3.2. Exosome Biogenesis

**Figure 2:**
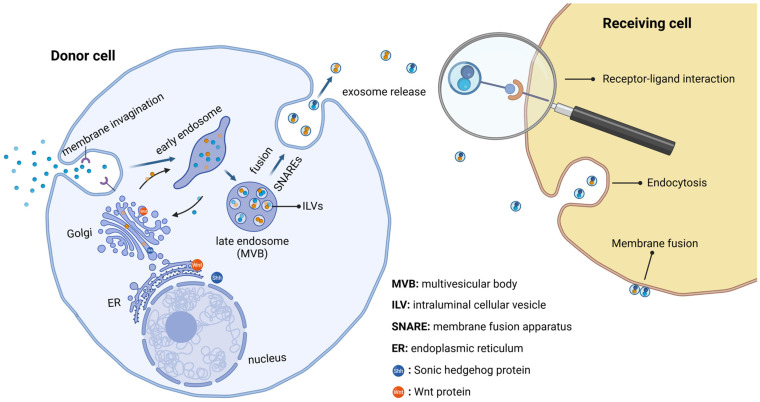
Graphical presentation of exosome biogenesis, release and uptake from receiving cells. Shh and Wnt proteins can also be transported in this way. Created in BioRender.com/m11g115.

Genesis of exosomes occurs in three main stages. These stages are exosome forming, sorting of cargo into exosomes, and exosome release [29] (Figure 2). More specifically, at first, the cytoplasmic membrane invaginates, and early endocytic vesicles are shaped. The early endosomes then invaginate, engulfing intraluminal cellular vesicles (ILVs). ILVs are transformed into late endosomes or multivesicular bodies (MVBs). MVBs fuse with the cytoplasmic membrane and ILVs are secreted in the extracellular space as exosomes [29].

Exosome biogenesis and secretion require the formation of an endosomal sorting complex. ESCRT machinery includes four protein components, namely ESCRT-0, ESCRT-I, ESCRT-II, ESCRT-III, and the associated proteins VPS-4, TSG101 and ALIX. ESCRT-0 sorts the protein cargo in the lipid domain, ESCRT-I and ESCRT-II promote membrane invagination in order to generate the membrane neck (an early stage of exosomal formation), while binding of VPS–4 to the ESCRT-III complex leads to vesicle neck disruption and recycling of the ESCRT-II complex [29].

An abundance of studies have revealed an ESCRT machinery-independent pathway of EV formation and cargo profile. This mechanism suggests that lipids and associated proteins play a key role in exosome biogenesis [30]. In addition, specific RNA sequences are found to display increased affinity to lipid rafts [31,32]. Ceramide, a lysophospholipid and glycosylsphingolipid molecule on the membrane, promotes membrane budding and the subsequent formation of ILVs [33]. Ceramidase and sphingosine kinase catalyze the conversion of ceramide to sphingosine and sphingosine I phospate (SIP). This reaction facilitates the tetraspanin sorting in ILVs [34]. Studies in mammals revealed that the abrogation of ESCRT machinery does not block the biogenesis of MVB, but this inhibition leads to diminished cargo shorting in ILVs [35]. It is proposed that exosome formation could be a crosstalking operation which encompasses ESCRT-dependent and ESCRT independent pathways [35].

### 3.3. Exosome Secretion

Exosome secretion depends on several critical factors, such as GTPase molecules, cytoskeleton microfilaments, molecular motors (dynein, kinesin) and the membrane fusion apparatus (SNARE complex) [36]. Rab-GTPases are crucial for exosome intracellular transport; they include 70 subclasses of proteins and adjust vesicle transport processes such as budding, motility and fusion [37]. The cytoskeleton microfilaments promote intracellular delivery of MVBs, which leads to different concentrations within the cells. The exact procedure of MVB fusion with the cell membrane is unclear, although SNAREs, the protein family of soluble N-ethylmaleimide-sensitive factor attachment protein receptors, has been considered as the key player [38]. The conjunction of vesicle SNAREs (v-SNAREs) with the target membrane SNAREs (t-SNAREs) drives the SNAREs complex formation, which leads to the fusion of MVBs with the plasma membrane and, thus, exosome secretion [38] (Figure 2).

### 3.4. Exosome Transport and Uptake

Exosome cargo can be transported to target cells via three district mechanisms, which include (a) endocytosis, (b) direct membrane fusion, and (c) receptor–ligand interaction [39]. Recent studies report that endocytosis is the most important process for exosome uptake by receiving cells. Exosomes can be incorporated by caveolin-, clathrin-, and lipid raft-arbitrated endocytosis. After that, exosomes may meld into endosomes or may be degraded by lysosomes [40]. The exosomal membrane can also fuse with the plasma membrane in order to deliver exosomal content into receiving cells. Exosomes can also bind to the target cell plasma membrane via ligand–receptor interaction [41], and then activate intracellular signaling pathways (for example the AKT pathway). EVs containing Hh and Wg components were first observed after biochemical degradation of naïve Hh and Wg expressing cultured insect cells [42] (Figure 2).

### 3.5. Morphogens Are Transported by Exosomes

The biochemical analysis of these vesicles highlighted GTPases of the Rab superfamily as critical players in EVs biogenesis [43]. Abrogation of these protein molecules resulted in reduced release [44] of morphogens in the extracellular space. Simultaneously, isolated Shh- and Wnt-containing vesicles are found to have the same biophysical features as exosomes, like density and zeta potential [44]. The conclusion is that overexpressed Shh and Wnt components are secreted through EVs in cultured cells [45]. In the Drosophila wing imaginal disc, released Shh and Wnt proteins were both detected far away from the secretion site and were bound to exosome markers (Shh associated with ESCRT [46] or the tetraspanin CD63—GFP and Wnt proteins with CD63-GFP and Rab-4 protein) [43,47]. Controlled depletion of ESCRT machinery leads to an accumulation of Shh on the cell surface and a decrease in long-range Shh activity [46]. Endogenous secreted Shh and ESCRT subunits, such as CHAMP-1, were identified on the membrane of Shh-receiving cells, concluding that these proteins are transferred together to reach receiver cells [46].

Previous studies demonstrated the existence of two pools of Shh-containing EVs with distinct roles. However, the way morphogens that are transported on EVs bind to their receptors is unclear. It has been proposed that Shh protein located on EVs binds to its receptor, Patched-1 (PTCH-1), at the surface of target cells. The overexpression of a mutant molecule of PTCH that cannot be endocytosed in the wing disc results in the trapping of Shh bound to ESCRT–CHAMP-1 particles, confirming that PTCH receptor interacts with Shh-containing EVs. This interaction leads to exosome fragmentation [46].

### 3.6. Wnt Signaling Pathway Components Transport by Exosomes

The Wnt palmitoylation facilitates the association of Wnts to FZD receptors [48], confirming that Wnts must be liberated from EV-membrane in order to function as signaling molecules. A novel study suggested that Wnt is liberated from the surface of EVs. This cleavage is mediated by the ADAMTS family protein Sol narae, leading to the generation of an active C-terminal Wnt fragment [49]. Expression of this cleavage product is sufficient to promote the expression of low-threshold Wnt target genes in Drosophila wing imaginal disc. In rats, Hedgehog Inhibitor Protein (HIP), a Sonic hedgehog (Shh) antagonist, is secreted on EVs from injured endothelia cells provoked by acute graft versus host disease (a GVHD) [50]. HIP associated with Shh on EV inhibits the endothelial repair by the Shh signaling pathway [51]. Other Shh pathway components have been also observed on EVs. For instance, cervical cancer cells and Drosophila wing disc cells secrete EVs that contain Shh protein receptors PTCH1 homolog 1 and Smoothened homolog SMO [52].

Shh and Wnt distribution via EVs is regulated by filopodia structures called cytonemes [53]. The Shh- and Wnt-containing EVs travel along cytonemes, and are released from cytonemes or bud from their tips [53]. Many studies revealed that EVs transmit Shh, Wnt proteins and also signal transduction pathway components, which regulate the morphogens’ activity.

### 3.7. Regulators of Lipid-Modified Morphogens Transport via EVs

The secretion of PTCH-1 via EVs promotes the formation of the Shh gradient in the wing imaginal disc [52]. EVs have also been found to transport regulators of the Wnt signaling pathway (e.g., β-catenin and FΖD) [54]. A mutated form of β-catenin with several gain-of-function properties was identified in EVs secreted from the colon cancer cell line LIM12135 [55]. In another study, the inhibitor of Wnt signaling pathway proline-rich protein 7 (PRP-7), was detected in EVs released from mouse hippocampal neurons [56]. Increased expression of PRP-7 in hippocampal neurons leads to the inhibition of the synaptic formation by Wnt5a and Wnt7a. Additionally, RNAi-induced blocking of PRP-7 promotes an increase in the concentrations of Wnt5a and Wnt7a in exosomes released from cultured rat hippocampal neurons. Increased Wnt signaling pathway activity leads to decreased concentration of PRP7 on EVs, confirming a Wnt-signaling-dependent feedback mechanism that governs the activity and release of Wnts on EVs [56]. The Wnt chaperone protein Wls is also released in EVs at synapses in Drosophila [42].

## 4. Other Transporters of Hedgehog Morphogen Components and Wnt/β-Catenin Proteins

### 4.1. Lipoproteins as Transport Vehicles of Lipid-Modified Morphogens

The first observation that Wnt components may be carried by lipoproteins was the association of membrane-tethered GFP with Wnt-containing vesicles. These vesicles originated from the basolateral membrane of Wnt-secreting cells [57]. These particles (previously named argosomes) have been demonstrated as lipoproteins (structures that facilitate the transport of hydrophobic lipids and proteins). Lipoproteins are important for Wnt signaling, as evidenced by the co-isolation of Wnt with lipophorins upon electrophoresis. Abrogation of lipoproteins lowers Wnt gradients, as concluded by the decreased expression of target genes in Wnt target cells [57].

Similar results have been reported in Shh signaling, which is also a palmitate-modified morphogen. These observations demonstrate that lipoproteins are carriers of morphogens, in order to establish long-range signaling activity [58]. This conclusion has also been reported in mammalians, where Wnt3a binds to lipoproteins in the media of rat fibroblasts [59]. Wnt5a is secreted in the murine choroid plexus and it is essential for the morphogenesis of the dorsal hindbrain. It was found that Wnt5a is bound with lipoproteins in the choroid plexus and stimulates the hindbrain progenitor in which Wnt signaling components and receptors for lipoprotein particles have been identified [60].

Many cell groups are capable of lipoprotein synthesis. Wnt3a release through lipoprotein structures is found in intestinal endothelial cells where Wnt3a is co-isolated with apoB100 (an apolipoprotein bound to low-density lipoprotein molecules). Remarkably, Wnt3a is bound with both high-density (HDL), and low-density lipoproteins (LDLs); however, only HDL allows the secretion of Wnt3a from mouse fibroblasts [59].

### 4.2. Heparan Sulphate Proteoglycans (HSPGs)

Another suggested mechanism of morphogen transport is the interconnection of Wnt proteins with heparan sulphate proteoglycans (HSPGs), a structural part of the extracellular matrix. HSPGs associate with many ligands, and are considered to function as co-receptors to facilitate the association of ligands with the corresponding receptors. HSPGs have also been found to crosstalk with many morphogens [61], and are considered to induce Wnt biodistribution via ligand stabilization. HSPGs are also proposed to favor the association of Wnt with its receptor, because overproduction of Wnt can establish the normal tissue patterning of the mutant Drosophila, which is deficient in an essential enzyme for proteoglycan synthesis [62].

HSPGs are components of the cell surface and the extracellular matrix that facilitate crucial interaction between cells and extracellular space, while they also modulate the delivery of morphogens and chemokines. FGF was the first growth factor found to depend on heparin sulphate for interaction with its receptor. Later, this observation was expanded to other pathways, namely the Shh, BMP, and Wnt/β catenin pathways [61]. In other organisms, like Zebrafish, the HSPG-encoding gene glypican 4 controls the gastrulation procedure through stimulation of Wnt 2 signaling [63]. In Xenopus embryos, glypican 4 interplay with WntII [64] and XEXT-1 (a glycosyltransferase that participates in heparan sulphate biogenesis) is critical for Wnt 2–induced neural axis shaping [64].

### 4.3. Soluble Forms of Lipid-Modified Morphogen Transport

It is known that ADAM-17 (a disintegrin and MMP domain protein) is implicated in the biogenesis of a lipid-free soluble and signaling component of cultured cell lines [65]. ADAM-17 cleaves lipid moieties, such as cholesterol and palmitate molecules, from Hh components, but also removes the CW domain from the N-terminal of Shh proteins [66]. ADAM-17-induced lipid-free Shh is biologically sufficient in promoting osteoblast differentiation and chick chondrocyte shaping in vitro. It is elusive if these lipid-free soluble components of the pathway favor tissue patterning in vivo [66]. The cholesterol- and palmitate-free Shh proteins are not adequate to travel at long distances because of the lack of the CW domain, which is essential for interplay between morphogen and HSPGs. However, soluble Shhh morphogens are identified in Drosophila wing discs [67]. This Shh is a cholesterol-free but palmitate-harboring Shh protein [68], named HhN+. HhN+ is different from the ADAM-cleaved lipid-free form, as was confirmed by electrophoresis. Releasing HhN+ in the hemolymph of Drosophila was aided by the location of Shh in the fat and the abrogation of lipoprotein biogenesis. HhN+ is dimeric/monomeric and signaling-adequate. Multimeric forms of Shh protein are released from Drosophila salivary grants for long-range signaling [68].

A group of proteins are reported to associate with Wnts. These proteins are called frizzled-related proteins (sFRPs) [69], and they regulate Wnt signaling via crosstalking with Wnt receptors or fragmentation of Wnt proteins. sFRPs may act as Wnt inhibitors or Wnt signaling inducers in a concentration-dependent manner [69]. They have been reported to stimulate the biodistribution of Wnt8 and Wnt II by building a complex [69,70], while the secreted Wnt interacting molecule (a lipocalin protein) was proposed to augment long-distance Wnt delivery by promoting its solubility [71]. However, the swim homologue has not been observed in vertebrates.

Afamin is a member of the albumin family of proteins, along with serum albumin, a-fetoprotein, and vitamin D-binding protein, with a high affinity for many lipid-reshaped proteins and other poorly soluble molecules. The afamin family has been identified to bind to Wnt proteins [72], promoting Wnt3a biological function by potentiating its solubility [73]. The biological activity of the afamin–Wnt3a complex was observed in intestinal organoid culture. Specifically, active Wnt3a signaling is required for the maintenance of the stem cell population which is located at the bottom of intestinal crypts.

This albumin is secreted by hepatocytes and is not an evolutionary conserved mechanism for Wnt distribution (invertebrates do not secrete afamin) [74]. Some studies report that Wnt proteins may be transported in the extracellular space with protein chaperons, something that can be visualized through fluorescence microscopy [75].

## 5. Cytonemes

### 5.1. Cytoneme Definition and Biogenesis—Basic Components of Morphogens on Cytonemes

Cytonemes are defined as actin-based dynamic cell-membrane projections that emanate from morphogen-producing or -receiving cells [53]. They function as molecular paths for the direct transport of developmental signaling molecules, cytokines, morphogens, and growth factors [76]. Their diameter is about 200 nm, and their length ranges from 4 to 70 mm [53]. Cytoneme biogenesis is mediated by activation of cytoskeleton inducers such as small GTPases [77], which promote actin remodeling. Activation of the β-catenin independent PCP pathway promotes activation of the small GTPases and thus favors membrane protrusions [78].

In Drosophila, the Wnt signaling pathway receptor FZD is located at the cytonemes of wing disc myoblasts. In these myoblasts, Wnt components are associated with FZD [76]. High-resolution imaging studies in Zebrafish also support the distribution of signaling molecules by cytonemes. The role of cytonemes is critical for Wnt transport in vertebrates too [79], since the ligand Wnt is carried via cytonemes to receiving cells [79]. Specifically, Wnt 2b—GFP and Wnt 8a—GFP have been identified on cytonemes in Xenopus and Zebrafish embryos, respectively [80]. The formation of Wnt-positive cytonemes depends on the expression of Wnt genes, while Wnt proteins are thought to travel from the ER to the plasma membrane with its chaperone protein WLS [81].

### 5.2. The Intestinal Crypt Paradigm

The Ror2 receptor has been observed as a non-canonical co-receptor for Wnts [82]. The binding of Wnt8a to Ror2 has been found to lead to the biogenesis of cytonemes by promoting actin polymerization [83]. Wnt proteins are considered to direct their traveling from secreting cells in this manner because both Ror2 and Wnt-8a concentrations are analogous to cytoneme expression. The mutant Ror2 leads to a decreased number of membrane protrusions in Zebrafish embryos [83]. Regulation of Wnt also influences Wnt–mediated proliferation of gastric cancer cells [84]. Cytonemes are also found in the intestinal crypt in the mucosa of the small intestine in mice [85]. The association of cytonemes with morphogen-mediated signaling and their sensitivity to cytochalasin treatment (the fungal metabolite that binds actin and prevents its polymerization) open new avenues in explaining normal tissue homeostasis and cancer behavior [85].

The neighboring cells to the intestinal crypt (stromal cells) release Wnt components [85]. Cytoneme size can be modulated by HSPGs. Abrogation of glypicans Dally and Dlp has a negative impact on the length of cytonemes, and the filaments are absent in Dally/Dlp double mutants [86]. Considering that Hh is also a lipid-modified morphogen, a cytoneme-based distribution may be suggested [86].

### 5.3. Exosome Movement Along Cytonemes

**Figure 3:**
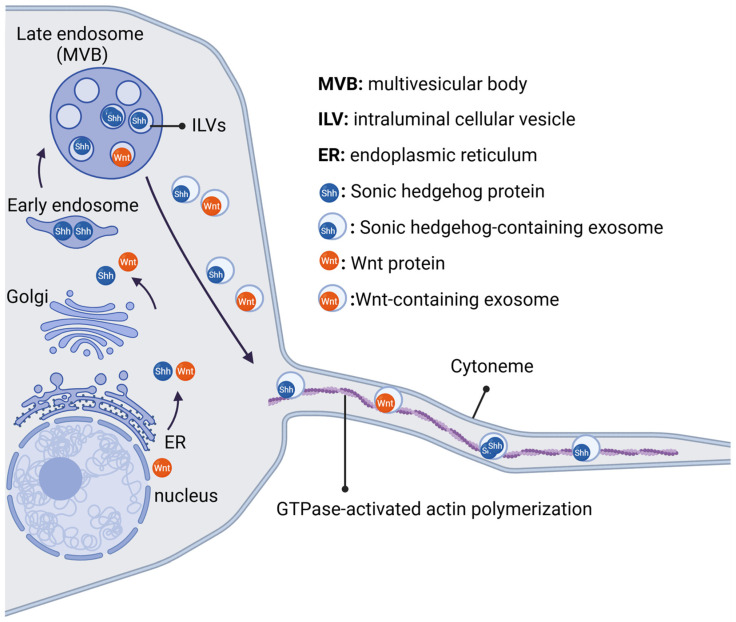
Graphical presentation of exosome movement along cytonemes. Created in BioRender.com/m59m083.

In Drosophila, the Shh signaling components on cytonemes are detected as spots that move along the cytoneme [53]. Their volume and the demonstrated co-placement of Shh and its co-receptor Ihg with the exosomal biomarker CD63–GFP establish their characterization as exosomes [47]. Wnts are also placed in association with exosomal biomarkers, and the forming Wnt spots have been reported on cytonemes [47]. A cross-talking between exosomes and cytonemes has been detected in the transport of exosomes to receiving cells, as exosomes move along the cytonemes [47]. Interaction between exosomes and cytonemes is possible, and may be a cooperative option for Wnt trafficking [53] (Figure 3).

## 6. Shh- and Wnt-Containing EVs—Implication for Diseases

Shh-containing EVs have been implicated in many diseases. EVs containing Shh are secreted into blood circulation after T-cell activation or apoptosis [87]. These EVs stimulate the nitric oxide (NO) pathway and contribute to the restoration of the endothelium damage. They also induce the expression of vascular endothelial growth factor VEGF [88,89], and thus participate in the angiogenesis procedure. Microparticles that carry Shh increase mRNA and protein levels of proangiogenic factors, as estimated by quantitative reverse transcription polymerase chain reaction. Additionally, these effects were reversed after abrogation of the shh receptor using RNAi technologies or when Shh signaling was inhibited by cyclopamine [89].

Shh has been also identified on EVs released by hepatocytes that accelerate liver remodeling in cirrhosis [90]. During hepatic injury, the myofibroblastic hepatic stellate cells and ductal-type progenitor cells release microparticles that contain Shh ligands that induce Shh-dependent changes in sinusoidal gene expression, and result in capillarization and the release of vasoactive factors which promote vascular remodeling [90].

Studies using rat models of ischemic injury demonstrated that Shh EVs have a remedial role and angiogenetic action [91]. Shh released in epithelial hair follicle cells favors the formation of the embryonic dentate gyrus in the mouse hippocampus [92]. The Shh precursor molecule is loaded on EVs and is distributed to the dentate gyrus of the hippocampus through the blood–brain barrier [92]. Wnt-enclosing EVs are secreted from neurons of Caenorhabditis elegans. The inhibition of these EVs’ formation results in behavior disorders [93].

In Drosophila’s neuromuscular space, the Wnt-enclosing EVs favor post-synaptic sensitivity [94]. In vertebrates, Wnt-3a is a hematopoietic growth factor that promotes acute myeloid leukemia (AML) clone expansion [95]. Using a methylation-specific polymerase chain reaction, aberrant methylation of Wnt /β catenin was observed in four AML cell lines and up to 64% of AML marrow samples. Treatment of these cell lines with aza-2-deoxycytidine promotes re-activation of Wnt inhibitors and suppression of Wnt signaling pathway target genes Cyclin-D1, TCF1 and LEF1. The hypermethylation of Wnt inhibitors is related to decreased 4-year relapse-free survival [95].

Wnt3a-containing EVs restore the proliferation of the side population of B-cell lymphoma cells in a clonogenic pattern with stem cell-like features [95]. Wnt-carrying EVs induce fibrotic tissue development within the myocardium and subsequent alteration of the heart’s normal architecture [96]. Recent studies have reported that such EVs isolated from L-cells (fibroblasts of mouse subcutaneous connective tissue) can activate the canonical Wnt pathway in cultured human fibroblasts [96]. In the human umbilical cord’s mesenchymal stem cells, Wnt-4-loaded EVs stimulate B-catenin in neighboring endothelial cells and induce a proangiogenic procedure [97].

In a mouse skin-injury model, EVs harboring Wnt-4 components promote epithelization [98]. Hu-MSC EVs promote wound healing by delivering Wnt-4 in order to activate Wnt/β-catenin in skin cells and subsequently inhibit acute heat-stress-induced skin cell necrosis via activation of the AKT pathway. Allogenic hu-MSC-derived exosomes present an alternative approach for stem cell-based therapy for skin injury repair [98].

Wnt-4 release on EVs derived from cancer cells favors angiogenesis and stimulates tumor development [99]. Previously, hypoxic and normoxic exosomes were injected into the center of a xenograft tumor model in rats. The hypoxic exosome treatment promoted tumor growth, compared to the normoxic one. The immunostaining analysis of tumor tissues with endothelial marker CD31 revealed that the CD31+ cells were increased in the hypoxic exosome-treated tumor cell group [99]. Macrophage-released EVs harboring Wnts have been found to play a leading role in maintaining the structural integrity of the intestinal endothelium. They are also required following injury; for instance, Wnt7b is required for kidney endothelial repair [100]. Macrophage Wnt signaling components are required for stem cell regeneration in the liver following damage to this organ [101]. The role of Wnt-5a bound to EVs in vitro is a subject of intensive research in cancer cell metastasis [102]. EVs purified from lung adenocarcinoma cell culture trigger Wnt signaling and induce tumor growth and metastasis in mice with adenocarcinoma [102]. Fibroblasts in a breast cancer microenvironment secrete Wnt ligand-containing microvesicles, which are internalized by breast cancer cells. These microparticles in turn promote the invasive behavior of breast cancer [102].

Exosomes containing Wnt components are observed in the hematopoietic microenvironment in health and disease. They participate in the development of multiple myeloma bone disease [103]. In healthy adults, bone remodeling is executed to replenish old bones and to strengthen bone tissue in response to physical stress or at sites of injury [104]. Osteoclasts resorb the damaged bone tissue, and then tissue-specific macrophages clean the compromised area. Osteoclasts recruit and activate osteoblasts to form new bone tissue, in order to fill the created space. Bone-forming osteoblasts secrete Wnt5a, which is a ligand for Ror2 receptors that are present on the surface of the osteoclast precursors [104]. Subsequent activation of the downstream signaling cascade stimulates differentiation into more mature bone-resorbing osteoclasts [105]. Thus, Wnt signaling influences RANK/RANKL/OPG axis signaling. The receptor RANK, which is expressed on the plasma membrane of the osteoclasts, can be activated by binding of its ligand RANKL that is expressed by osteoblasts. In addition, osteoblasts secrete OPG, a natural decoy receptor that antagonizes with RANK for the binding of RANKL [104]. On the other hand, osteoclasts regulate osteoblast action by secreting WNT10b [104,105]. Multiple myeloma cells induce osteolytic bone disease by activating osteoclasts and inhibiting osteoblasts. They promote osteoclast function by secreting RANKL [105], and they inhibit osteoblasts by secreting Wnt inhibitors FRP-2 and DKK. Both genes (RANKL and OPG) are target genes of the Wnt signaling pathway [104]. Increased Wnt signaling activity leads to high secretion of OPG and low activity of RANKL, which subsequently results in decreased osteoclastogenesis [104].

## 7. Conclusions

Cell-derived structures (exosomes and cytonemes) play a significant role as components of the communication between cells in order for tissue patterning to be established. On the other hand, morphogenesis is a critical process in the establishment and maintenance of tissue polarity shaping and microarchitecture.

Although the present review underlines the role of EVs in tissue patterning as critical, there are still open questions. For instance, the way EVs influence the transcriptional process in the early phases of embryonic development, as well as their potential to filter morphogen signals that participate in tissue forming and organ shaping, must be determined. Wnt and Hh morphogens belong to the great family of non-classical transcription factors. They undergo lipid modification (palmitoylation and cholesterol-adding), which is necessary for their free diffusion but which makes the long-range distribution and action of lipid-modified morphogen impossible. The mechanism that ensures long-range travelling and the action of lipid-modified morphogens is their incorporation in EVs. Finally, the application of morphogen-containing EVs in the therapeutic field of genetic diseases, in regenerative medicine, and as a target treatment in various malignancies remains to be clarified in the future. This may be achieved by utilizing the advantages of exosomes, namely, tumor-tissue homing ability, extended blood circulation, hypo-immunogenicity, low toxicity, and excellent blood–brain barrier traversal.

## Data Availability

Not applicable.

## Abbreviations

The following abbreviations are used in this manuscript:

ADAM-17	Disintegrin and metalloprotein domain-containing protein 17
AKT	Protein kinase B
ApoB100	Apolipoprotein bound to LDL molecules
BMP	Bone Morphogenetic Protein
CHAMP-1	CHromosome Alignment-Maintaining Phosphoprotein 1, subunit of ESCRT
D.K.K.1	Dickkopf WNT signaling pathway inhibitor
Dlp	Dally-like protein
ER	Endoplasmic Reticulum
ESCRT	Endosomal, Sorting, Complex, Required for Transport
EVs	Extracellular Vesicles
FGF	Fibroblast Growth Factor
FZD	Frizzled protein
GFP	Green Fluorescent Protein
GLI	Glioma-associated oncogene homolog
GTP	Guanosine-5′-Triphosphate
HDL	High-Density Lipoprotein
HIP	Hedgehog Interacting Protein
HSP 90	Heat Shock Protein 90
HSPGs	Heparan sulphate proteoglycans
Hu-MSC	Human Umbilical Mesenchymal Stem Cells
IGF-1	Insulin-like Growth Factor 1
ILVs	Intraluminal cellular Vesicles
LDLs	Low-Density Lipoproteins
LEF1	Lymphoid Enhancer Binding Factor 1
LRP	Low-density lipoprotein Receptor-related Protein
LRP5/6	Low-density lipoprotein Receptor-related Protein 5 and 6
MHC	Major Histocompatibility Complex
MVB	Multivesicular Bodies
OPG	Osteoprotegerin, natural decoy receptor for RANKL
PCP pathway	Planar Cell Polarity pathway
PI3K/AKT	Phosphoinositide 3-Kinase (PI3K)/protein kinase B (AKT)
PRP-7	Proline-ich Protein-7
PTCH-1	Patched 1 protein
RANK	Receptor Activator of Nuclear factor Kappa-Β
RANKL	Receptor Activator of Nuclear factor Kappa-Β Ligand
RNA	Ribonucleic acid
Ror2	Receptor tyrosine kinase-like orphan receptor
sFRPs	Secreted Frizzled-related proteins
Shh	Sonic hedgehog
SIP	Sphingosine-1-Phosphate
SMO	Smoothened–a G-protein-coupled receptor
SNAREs	Soluble N, Ethylmaleide sensitive factor Attached protein Receptors
SUFU	Suppressor of Fused homolog
SWIM	Secreted Wg Interacting Protein
TCF1	T-Cell Factor 1
TCF/LEF	T-Cell Factor /Lymphoid-Enhancing Factor
TGF-1	Transforming Growth Factor-1
TGF-B	Transforming Growth Factor-B
TSG101	Tumor Susceptibility Gene 101 protein
VEGF	Vascular Endothelial Growth Factor
VPS	vacuolar protein sorting-associated protein
Wls	Wnt ligand secretion mediator
Wnt	Wingless-related integration site
XEXT	glycosyl transferases of the exostosin family
